# Removal of Copper from Water by Adsorption with Calcium-Alginate/Spent-Coffee-Grounds Composite Beads

**DOI:** 10.3390/ma12030395

**Published:** 2019-01-27

**Authors:** Roberto Torres-Caban, Carmen A. Vega-Olivencia, Luis Alamo-Nole, Daisy Morales-Irizarry, Felix Roman-Velazquez, Nairmen Mina-Camilde

**Affiliations:** 1 University of Puerto Rico, Mayaguez Campus, Department of Chemistry, Mayaguez 00681, Puerto Rico; carmenamaralis.vega@upr.edu (C.A.V.-O.); felixr.roman@upr.edu (F.R.-V.); nairmen.mina@upr.edu (N.M.-C.); 2 Pontifical Catholic University of Puerto Rico, Ponce Campus, Tosello Giangiacomo Scientific Research Center, Ponce 00731, Puerto Rico; luis_alamo@pucpr.edu (L.A.-N.) moralesdaisy@pucpr.edu (D.M.-I.)

**Keywords:** removal of copper, calcium alginate, spent coffee grounds, water pollution, kinetics of adsorption

## Abstract

Calcium Alginate/Spent-Coffee-Grounds composite beads (CA-SCGs beads), which were made of two different proportions of alginate and spent-coffee-grounds (3:3 and 3:10), respectively, were used to adsorb Cu^2+^ in aqueous solution. These beads were compared with calcium alginate beads (CA beads) and spent-coffee-grounds (SCGs) in terms of adsorption capacity and rate of adsorption. The experiments were carried out at an initial pH of 4 at 30 °C with initial concentrations of Cu^2+^ from 10 ppm to 100 ppm. Equilibrium data was fitted with Langmuir, Freundlich and Sips models, and a pseudo-second-order kinetic equation. The Sips model showed the best correlation with the experimental values. CA-SCGs (3:3) beads showed a faster adsorption rate versus the CA beads. Also, CA-SCGs (3:3) beads showed a larger capacity of adsorption according to the Sips model, but not in the Langmuir model. FT-IR spectra and SEM images were taken for characterization. This study has shown that the CA-SCGs (3:3) beads have a synergistic effect, combining the capacity of adsorption of CA beads with the kinetics of the SCGs. The CA-SCGs beads have proven to be an effective adsorbent of Cu^2+^. Therefore, they can provide a use for the SCGs; which are considered pollutants in landfills.

## 1. Introduction

Copper is primarily used as the metal or alloy in the manufacture of wire, sheet metal, pipe, and other metal products. Additionally, compounds of copper are used in agriculture to treat plant diseases. Copper is frequently found near mines, smelters, industrial settings, landfills, and waste disposal sites. In the case of copper and copper compounds that have been released into the water, the copper that dissolves can be carried in surface waters either in the form of copper compounds or as free copper or, more likely, copper bound to particles suspended in the water. Humans could be exposed to soluble copper levels in drinking water above the acceptable drinking water standard of the United States Environmental Protection Agency of 1,300 parts per billion (ppb) if water has low pH and it is corroding copper plumbing [1]. Also, the negative effects of acid mine drainage can be observed in places where there are sulfide ores and sulfide-containing raw materials used to be mined [2]. This requires removal of copper in acidic conditions where the copper is most soluble. Copper is also one of the most toxic metals to a wide spectrum of marine life [3]. Copper concentrations between 1 and 10 μg/L (ppb) can seriously affect a large number of marine organisms. These concentrations have lethal effects on scallops, clams and isopods [4]. For these reasons, it is important to control the release of copper before the return of the wastewater to the environment. 

Activated Carbon (AC) is the most commonly known adsorbent to control the release of pollutants. It is an adsorbent with large porous surface area, controllable pore structure, thermo-stability and low acid/base reactivity. It has the capacity for removal of a broad range of organic and inorganic pollutants dissolved in aqueous media. Although it has benefits in its use in adsorption processes, the biggest challenge of its application is the cost and difficulties associated with regeneration. For the reasons explained before, many studies are evaluating the feasibility and suitability of natural, renewable, low-cost materials and even waste materials such as bamboo dust, peat, chitosan, lignin, fungi, moss, bark husk, chitin, maize, cob, pinewood sawdust, rice husk, sugar cane bagasse, tea leaves, sago waste, alginate or even coffee grounds, as alternative adsorbents in water pollution control [5].

Annually, the industry of coffee is responsible of the production of more than 2 billion tons of by-products such as spent coffee grounds (SCGs) and coffee silverskin (CS), but most of it is thrown away without further use [6]. The chemical composition of SCGs is based on hemicellulose, cellulose, fat, proteins, polyphenols, minerals and products formed by the Maillard reaction during the roasting process, such as melanoidins [6,7]. The high content of organic material makes SCGs a contaminant due to a great demand of oxygen to be degraded [8]. Melanoidins are polymeric macromolecules originated by the Maillard reaction and formed by interactions between carbohydrates (reducing sugars) and compounds characterized by a free amino group, such as amino acids. The melanoidins are one of the major components of coffee beverages, accounting for up to 25% of dry matter. These compounds have a relevant chelating ability [9]. The melanoidins can be linked to proteins in SCGs. The melanoidins and some of the other major components of the SCGs, namely polysaccharides cellulose and hemicellulose, and the polymeric compound lignin can be used to remove heavy metals from water because they have functional groups as –OH, –COOH, amino and phenolic groups which are able to adsorb heavy metals from aqueous solutions [9,10,11,12]. Untreated SCGs have been used for the removal of heavy metals from water [13]. The coffee spent grounds is pollutant by itself and most of them are wasted in landfills. When coffee grounds are dumped into landfills, they create methane, which is a greenhouse gas [14]. 

Alginic acid or Alginate, is a polysaccharide distributed widely in the cell walls of brown algae. Usually, the alginate is used as calcium alginate (CA) that is insoluble and created with the addition of an aqueous solution of sodium alginate to an aqueous solution of calcium chloride. During the reaction, Na^+^ is replaced with Ca^2+^ to create the insoluble calcium alginate (CA). This process allows for the entrapment of compounds as a method for the posterior delivery of the compound. Calcium alginate beads (CA beads) have been used for the sorption of heavy metals, due to its good affinity for the bivalent metal ions [15]. Alginic acid (AA) is a polysaccharide composed of β-D mannuronic Acid(M) and α-L-glucuronic acid (G), but only the G-blocks of alginate are believed to participate in intermolecular cross-linking with divalent cations (e.g., Ca^2+^) to form hydrogels [16]. 

The purpose of the use of calcium alginate (CA) in this study is as a way to encapsulate the spent coffee grounds (SCGs) in a bead, even though the CA has a greater metal adsorption capacity by itself than the SCGs. The SCGs have an advantage over the CA, which is that the adsorption of metal by SCGs is faster than the adsorption by CA. CA beads may achieve the equilibrium in hours [15,17], while the SCGs achieve the equilibrium in minutes [18]. The synergistic effect of both materials in the same bead can address the possible issues in the use of both materials, separately, in the real world. This is the objective of this study. Also, the combination of a natural and renewable material as the CA with a common organic waste material as the SCGs is beneficial because the SCGs as waste is a pollutant by itself; different to the CA that is cheap, but not a by-product waste of human consumption. The encapsulation of the SCGs in a bead makes it easier to handle the SCGs after of the adsorption of heavy metals.

The values of maximum adsorption capacity of the calcium alginate beads (CA beads) reported in the literature are variable, e.g., 88.95 mg/g [13], 42.7 mg/g [19] and 111.9 mg/g [17]. This variability is due to many factors in the synthesis of the beads that can affect the final product. These factors could be: the type of sodium alginate used, the concentration of sodium alginate used, the concentration of the calcium chloride solution used as crosslinker, the time of hardening, the conditions of drying, the addition of other reactants, among others. In the same way, the experimental conditions can be different. These variables are beyond of the scope of this study, because in this study, the calcium alginate (CA) is only the matrix of encapsulation. The analysis of the calcium alginate beads (CA beads) prepared in this study is only to see the performance of this matrix alone versus the composite beads (CA-SCGs beads) and the spent coffee grounds (SCGs) alone. The objective of this study is to see the performance of the CA-SCGs beads as adsorbent of Cu^2+^ in water and how this performance is not only due to the matrix used for encapsulation but the synergistic effect between the SCGs and CA.

## 2. Materials and Methods

### 2.1. Spent Coffee Grounds (SCGs)

Spent Coffee Grounds (SCGs) were obtained from Café Colegial (Mayaguez, Puerto Rico), the coffee shop of the campus. SCGs were collected and immediately put to dry at 50 °C for five days. After drying, SCGs were sieved through a #30 ASTM sieve (600 µm). Previous experiments of metal removal, where the employed coffee was only washed with deionized water, showed that this adsorbent continued to release soluble coffee material on subsequent exposure to aqueous solution [20]. This fact could be an obstacle in the adsorption by competing with the metal cations, but when the alkali-leached material was used, it released very little colored material [21]. Therefore, the SCGs was pre-treated with 0.1 M NaOH (Fisher Chemical, Fair Lawn, NJ, USA) to remove the soluble materials as described in Dávila-Guzmán, N. E et al. [18]. After of the pre-treatment with NaOH, the SCGs was washed repeatedly with deionized water until a value of pH close to 6 was achieved. Then, the SCGs was dried again at 70 °C for 24 h.

### 2.2. Preparation of the Calcium Alginate Beads and the Calcium Alginate/Spent-Coffee-Grounds Composite Beads

For the preparation of the CA-SCGs (3:3) beads, 3 g of the dried pre-treated SCGs were weighted. One hundred millimeters of deionized water were added to a beaker. A laboratory paddle stirrer was used to stir the solution to the maximum capacity. Three grams of SCGs were added to the water. After this, 3 g of sodium alginate (ACROS ORGANICS, Morris, NJ, USA) were added. Then, 100 mL of deionized water were added again to achieve a volume of 200 mL. This solution of 200 mL of deionized water had 3 g of sodium alginate (1.5% w/v sodium alginate) and 3 g of SCGs. This solution was used to make CA-SCGs beads (3:3). For the other beads, the only change was the amount of SCGs used. Ten grams of SCGs were used to make the CA-SCGs beads (3:10). The CA beads were prepared with the same procedure but without SCGs (3:0). The following step in the preparation of the beads is to add the solution with sodium alginate prepared above to the solution of 0.2 M CaCl_2_. 

A solution of 300 mL of 0.2 M CaCl_2_ (Fisher Chemical, Fair Lawn, NJ, USA) was prepared in another beaker. The solution of sodium alginate/SCGs was added dropwise to the solution of 0.2 M CaCl_2_. With each drop, a bead was formed. After all of the sodium alginate/SCGs solution was added to the 0.2 M CaCl_2_ solution, the new solution was maintained at constant stirring with a magnetic stirrer for 2 h more to harden the beads. Then, the beads were washed with deionized water and dried in an oven at 40 °C for four days. 

### 2.3. Adsorption Experiments and Kinetic Experiments

Working solutions of 100 mL of Cu^2+^ were prepared from a commercial stock solution of 1000 ppm (Sigma-Aldrich, Saint Louis, MO, USA). The concentrations of the working solutions were 10 ppm, 20 ppm, 30 ppm, 60 ppm, 80 ppm, and 100 ppm. The working solutions were prepared in triplicate for each concentration. The pH was adjusted to 4 with NaOH (Fisher Chemical, Fair Lawn, NJ, USA) and nitric acid (Sigma-Aldrich, Saint Louis, MO, USA). Each working solution was added to a plastic bottle of 120 mL for the experiment. Each bottle had a volume of solution of 100 mL and an amount of adsorbent of 0.5 g. The adsorbent could be SCGs, CA beads, CA-SCGs (3:3) beads or CA-SCGs (3:10) beads. The bottles were placed in an orbital shaker at 30 °C, at 250 rpm. Samples were taken at different periods of time 0 min, 30 min, 60 min, 90 min, 120 min (2 h), 180 min (3 h), 240 min (4 h), 360 min (6 h), 720 min (12 h), and 1,440 min (24 h). In the case of SCGs, since the adsorbent is a powder, not a bead, a small change was made in the sampling procedure to avoid a possible clogging of the instrument used to analyze the metal content. A sample was taken from the plastic bottle in the orbital shaker at the specific time, centrifuged for 5 min at 3200 rpm, then a specific volume from the supernatant was taken. The samples were diluted with 2% v/v nitric acid and refrigerated. The dilution factor was taken into consideration in all the samples. The samples were analyzed for copper content by inductively coupled plasma optical emission spectrometry (Pelkin-Elmer ICP-OES 3300XL, Perkin-Elmer, Billerica, MA, USA) at a wavelength of 224.7 nm. 

The adsorption capacity of Cu^2+^ at equilibrium (*q_e_*) was calculated using the following equation Equation (1) [22]:

*q_e_* = (*C*_0_ − *C_e_*)*V*/*W* (1)

where *C*_0_ (mg/L) is the initial concentration, *C_e_* (mg/L) is the equilibrium concentration; *V* is the volume of the solution of Cu^2+^ ions (L) and *W* is the weight of adsorbent added to the solution (g). The time of equilibrium was 1440 min (24 h). 

For the kinetic experiments, adsorption capacity of Cu^2+^ at time t (*q_t_*) was calculated with the following equation Equation (2) [23]:

*q_t_* = (*C*_0_ − *C_t_*)*V*/*W* (2)

where *C_t_* is the concentration of Cu^2+^ at time *t*.

### 2.4. Adsorbent Dose Effect (Beads)

The effect of different doses of CA beads, CA-SCGs beads (3:3) and CA-SCGs beads (3:10) in the adsorption of Cu^+2^ ion were measured at 0.025 g, 0.050 g, 0.150 g and 0.500 g in 0.1L at a concentration of 20 ppm of Cu^2+^ for 24 h as the equilibrium time.

### 2.5. Characterization

Fourier-transform infrared spectroscopy (FT-IR) spectra of the CA beads, CA-SCGs beads, pre-treated SCGs and raw SCGs was obtained using an Agilent Cary 630 FT-IR Spectrophotometer (Agilent, Santa Clara, CA, USA), Attenuated Total Reflection (ATR). Transmission spectra were obtained in the range of 4000–450 cm^−1^. An air background was used as a blank for all spectra collected. The most important peaks were identified at its specific wavenumber to see which peaks are characteristic of the calcium alginate, of the spent-coffee-grounds, or both. The purpose is to take notice of the presence or absence of functional groups in each adsorbent. To see the surface morphology and porous structure of the beads, a JEOL JSM-5410LV Scanning Electron Microscopy (SEM, JEOL Ltd., Peabody, MA, USA) was used at three different levels of magnification to see the changes in the surface morphology at each level. The sample was coated with a thin layer of gold before the analysis. The appearance of porosity and roughness of the surface was evaluated at three different levels of magnification. 

## 3. Results and Discussion

### 3.1. Characterization of CA Beads, CA-SCGs Beads, Pre-treated SCGs and Raw SCGs

**Figure 1:**
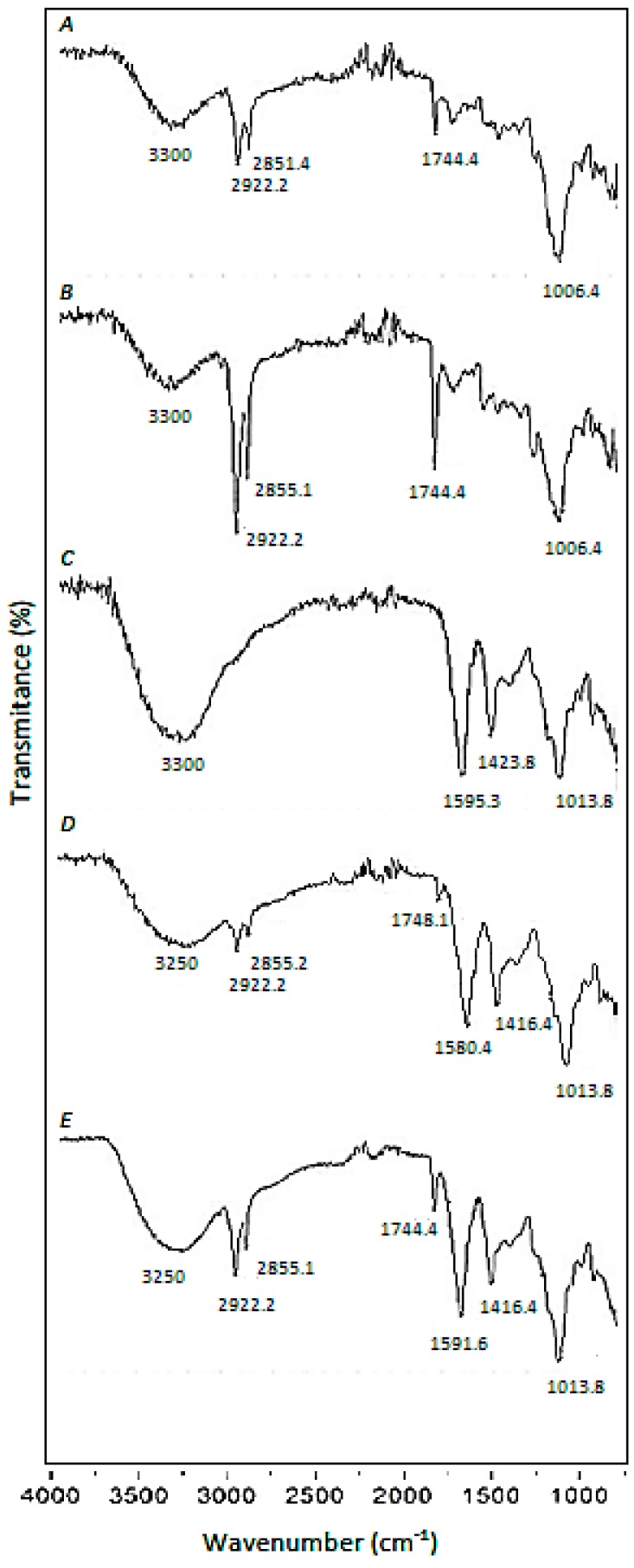
FTIR spectra. (**A**): Raw Spent-Coffee-Grounds (SCGs), (**B**): Pre-treated SCGs, (**C**): Calcium Alginate (CA) beads, (**D**): Calcium Alginate/Spent-Coffee-Grounds (CA/SCGs) (3:3) beads, (**E**): CA/SCGs (3:10) beads.

The broadband approximately at 3300 cm^−1^, which appears in all the spectra shown (Figure 1A–E), is mainly attributed to the presence of –OH groups in SCGs and CA, but in the case of SCGs (Figure 1A,B), it can include a minor contribution of –NH functional groups [24].

Three sharp bands at 2922 cm^−1^, 2855 cm^−1^, and 1744 cm^−1^ are characteristic of the SCGs (Figure 1A,B). They also appear in CA-SCGs (3:3) beads and CA-SCGs (3:10) beads (Figure 1D,E). The bands at 2922 cm^−1^ and 2855 cm^−1^ are attributed to asymmetric and symmetric stretching of C–H bonds in aliphatic chains and can be attributed to lipids that are present in large amounts in the SCGs [25]. The sharp band at 1744 cm^−1^ can be attributed to a carbonyl vibration (C=O) in aliphatic esters or in triglycerides, for this reason, this band is also attributed to lipids [25].

The CA beads have characteristic sharp bands at 1595 cm^−1^ and 1423 cm^−1^, which can be attributed to –COO^−^ (asymmetric stretching) and –CH_2_ (bending) [19]. These bands appear in CA-beads (Figure 1C), CA-SCGs (3:3) beads (Figure 1D), and CA-SCGs (3:10) beads (Figure 1E). The band related to 

–COO^−^ (1595 cm^−1^) also appears in SCGs, but with very low intensity.

Another sharp band is at 1013 cm^−1^; this band also appears in all the spectra that contain CA, SCGs or both (Figure 1A–E). This band at 1013 cm^−1^ can be attributed to C–O–C (stretching) vibrations [19,25].

The presence of –NH functional groups and lipids in the CA-SCGs beads can support the idea of a more heterogenous surface for adsorption by the CA-SCGs beads in comparison to the CA beads. This is because CA is a polysaccharide without lipids or amino groups.

**Figure 2:**
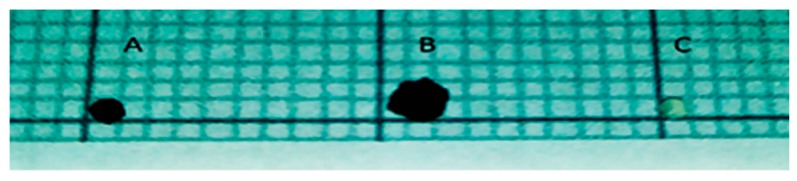
Photo of the beads: (**A**): CA-SCGs (3:3) beads, (**B**): CA-SCGs (3:10) beads and (**C**): CA beads. The diameters of the beads were: 1 mm (**A**); 2 mm (**B**) and 1 mm (**C**).

**Figure 3:**
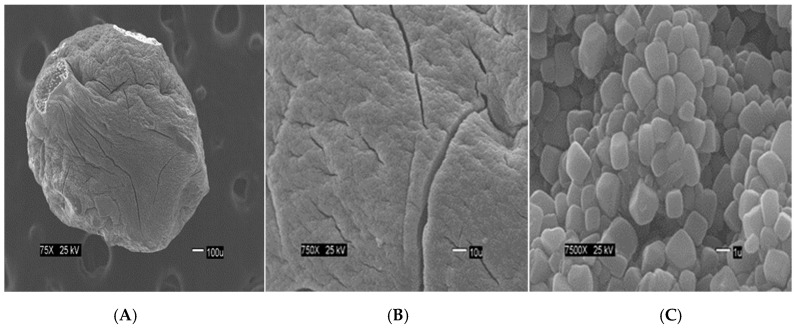
SEM images at three different magnitudes of CA beads: (**A**): 75×; (**B**): 750× and (**C**): 7500×.

**Figure 4:**
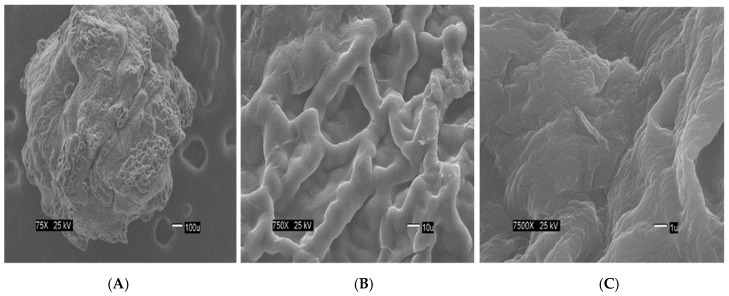
SEM images at three different magnitudes of CA-SCGs (3:3) beads: (**A**): 75×, (**B**): 750× and (**C**): 7500×.

**Figure 5:**
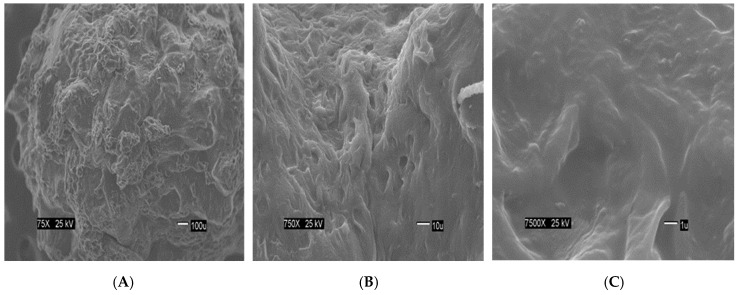
SEM images at three different magnitudes of CA-SCGs (3:10) beads: (**A**): 75×, (**B**): 750× and (**C**): 7500×.

The Figure 2 show the size of the beads. The SEM images show differences in the surface morphology of the beads. At the lowest magnification (75×), the surface of the CA beads (Figure 3A) seems flat with some cracks, but without much porosity. The surfaces of the CA-SCGs (3:3) beads (Figure 4A) and the CA-SCGs (3:10) beads (Figure 5A) look rough and porous. At an increment of 10 times the magnification (750×), the surface of the CA beads (Figure 3B) keeps the flat appearance, but with more visible cracks. At this magnification, the surface of the CA-SCGs (3:3) beads (Figure 4B) looks branched, while the surface of the CA-SCGs (3:10) beads (Figure 5B) does not look branched, but looks porous. At the highest magnification (7500×), the appearance of the surface of the CA beads (Figure 3C) is no longer a flat surface. At this magnification, the porosity in the surface of the CA beads can be seen. The surface of the CA-SCGs (3:3) beads (Figure 4C) loses its branching appearance, but some porosity and roughness can be still observed. The surface of the CA-SCGs (3:10) beads (Figure 5C) exhibits less roughness and porosity than the surface of the CA-SCGs (3:3) beads.

### 3.2. Adsorbent Dose Effect (Beads)

The effect of different doses of CA beads, CA-SCGs beads (3:3) and CA-SCGs beads (3:10) in the adsorption of Cu^2+^ ion was observed at 0.025 g, 0.050 g, 0.150 g and 0.500 g in 0.1 L at a concentration of 20 ppm of Cu^2+^ at 24 h as equilibrium time. It can be seen in the plot that the CA-SCGs beads (3:10) have a lower adsorption capacity (compared to the other beads) when the ratio of concentration of Cu^2+^ to the number of beads is larger, but the adsorption capacity improves when this ratio is smaller. The adsorption performance of the CA-SCGs beads (3:10) is due to their greater content of SCGs. A possible explanation of the adsorption performance at different adsorbent doses of these beads could be that SCGs have a lesser amount of adsorption sites than CA but these adsorption sites have a greater affinity to copper. The capacity of adsorption of the CA-SCGs beads (3:10) is dependent of the concentration of adsorbate. This fact is in accord with the Sips model [5].

### 3.3. Analysis of Adsorption Isotherms

An adsorption isotherm could be defined as an equilibrium relationship, since describes how pollutants interact with the adsorbent materials at a constant temperature. In a non-linear form, the isotherm equation is a relation between the amount of pollutant adsorbed at equilibrium versus the concentration of pollutant at equilibrium (*q_e_* vs. *C_e_*). The units of q_e_ are milligrams of pollutant per grams of adsorbent (mg/g). The units of *C_e_* are parts per million (ppm) of pollutant. A variety of equilibrium isotherm models (Langmuir, Freundlich, Brunauer–Emmett–Teller, Redlich–Peterson, Dubinin–Radushkevich, Temkin, Toth, Koble–Corrigan, Sips, Khan, Hill, Flory–Huggins and Radke–Prausnitz isotherm), have been formulated [26]. In this study, three models were used: Langmuir, Freundlich and Sips. 

Langmuir equation, the most used model of adsorption isotherm, assumes monolayer adsorption (the adsorbed layer is one molecule in thickness). The adsorption can only occur at a finite (fixed) number of definite localized sites that are identical and equivalent. No lateral interaction and steric hindrance between the adsorbed molecules. Langmuir isotherm refers to homogeneous adsorption, which each molecule possesses constant enthalpies and sorption activation energy, all sites possess equal affinity for the adsorbate, with no transmigration of the adsorbate in the plane of the surface. The non-linear Langmuir equation is described below, Equation (3) [15]:

*q_e_* = (*q_max_* × *b_L_* × *C_e_*)/(1 + *b_L_* × *C_e_*) (3)

where *q_max_* is the maximum monolayer coverage capacity (mg/g) and *b_L_* is the Langmuir isotherm constant (L/mg) related to the affinity. 

Freundlich equation can be applied to multilayer adsorption, with non-uniform distribution of adsorption heat and affinities over the heterogeneous surface. The stronger binding sites are occupied first, until adsorption energy is exponentially decreased upon the completion of adsorption process. The non-linear Freundlich equation is described below, Equation (4) [5]:

*q_e_* = *K_f_* × *C_e_*^1/*n*^ (4)

where *K_f_* is the adsorption capacity (L/mg) and 1/*n* is the adsorption intensity, which also indicates the relative distribution of the energy and the heterogeneity of the adsorbate sites, becoming more heterogeneous as its value gets closer to zero.

Sips equation is a combination of the Langmuir and Freundlich isotherms. The non-linear Sips equation is described below, Equation (5) [27,28]:

*q_e_* = (*q_max_* × *b_S_* × *C_e_*^1/*n*^)/(1 + *b_S_* × *C_e_*^1/*n*^) (5)

where *q_e_* is the adsorbed amount at equilibrium (mg/g), *C_e_* the equilibrium concentration of the adsorbate (mg/L), *q_max_* is the Sips maximum adsorption capacity (mg/g), *b_S_* is the Sips equilibrium constant (L/mg), and 1/*n* is the Sips model exponent. Values for 1/*n* < 1 indicate heterogeneous adsorbents, while values closer to 1.0 indicate a material with homogenous binding sites. If 1/*n* = 1, the Sips model is reduced to the Langmuir equation [27]. 

**Figure 6:**
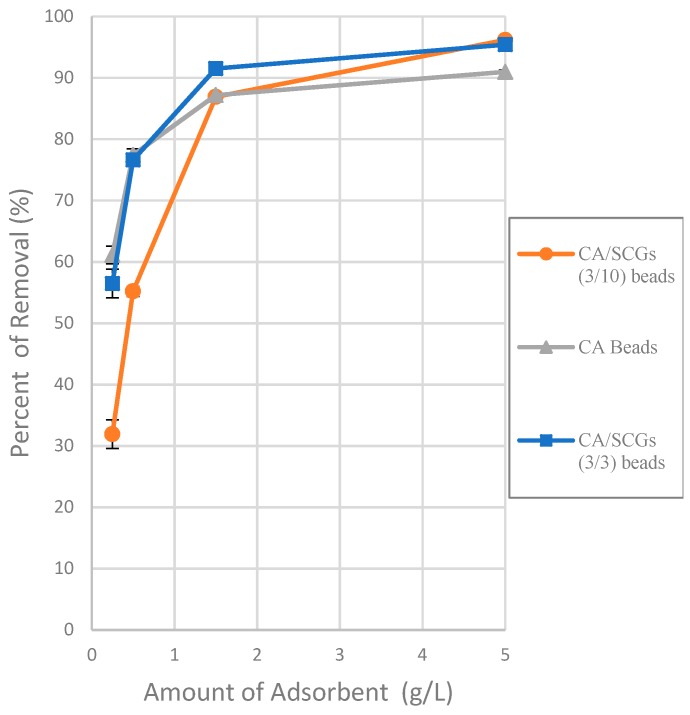
Plot of the effect of adsorbent dose on the adsorption of Cu^2+^ ions at a concentration of 20 ppm of Cu^2+^ at 24 h.

The Sips model is appropriate for predicting adsorption on heterogeneous surfaces while the model at the same time avoids the limitation of increased adsorbate concentration normally associated with the Freundlich model. At low adsorbate concentrations, the Sips model reduces to the Freundlich model, but at high concentrations of adsorbate, it predicts the Langmuir model (monolayer adsorption) [29]. For heterogenous surfaces, the problem of the continuing increase in the adsorbed amount with an increase in concentration in the Freundlich equation, is corrected by the Sips equation because it has a finite limit when the concentration is sufficiently high [30]. The parameters of the Sips isotherm are dependent of the concentration of adsorbate. This could explain the behavior of the CA/SCGs beads (3:10) in the plot of the Figure 6 (adsorbent dose effect). 

Non-lineal forms were used for the three models. All model parameters were evaluated by nonlinear regression using an optimization routine to minimize the sum of squared errors (SSE) in the concentration range used in adsorption experiments by the Microsoft Excel solver tool. This method seeks to minimize the sum of the squared errors (SSE) between observed and calculated values of the dependent variable. The sum of squared errors (SSE) was calculated from the following equation, Equation (6) [30]:

(6) $$\mathrm{SSE}=\overset{n}{\underset{i=1}{\sum }}{\left[q_{\exp}-q_{\mathrm{model}}\right]}^{2}$$

where q_exp_ is the experimental data, and q_model_ is the value predicted by the models at corresponding C_e_. In order to compare the applicability of isotherm equations and fitting it to the data; nonlinear correlation coefficient R^2^, relative error ∆*q* and nonlinear chi-square test χ^2^ were calculated.

Relative error ∆*q*, Equation (7) [27]:

(7) $$\Delta q\left(\%\right)=\left(1/n\right)\overset{n}{\underset{i=1}{\sum }}\left|\left(q_{\exp}-q_{\mathrm{model}}\right)/q_{\exp}\right|\times 100$$

Nonlinear chi-square test χ^2^, Equation (8) [31]:

(8) $$\chi^{2}=\overset{n}{\underset{n=1}{\sum }}[{\left(q_{e.\exp.n}-q_{e.\mathrm{model}.n}\right)}^{2}/q_{e.\exp.n}]$$

**Figure 7:**
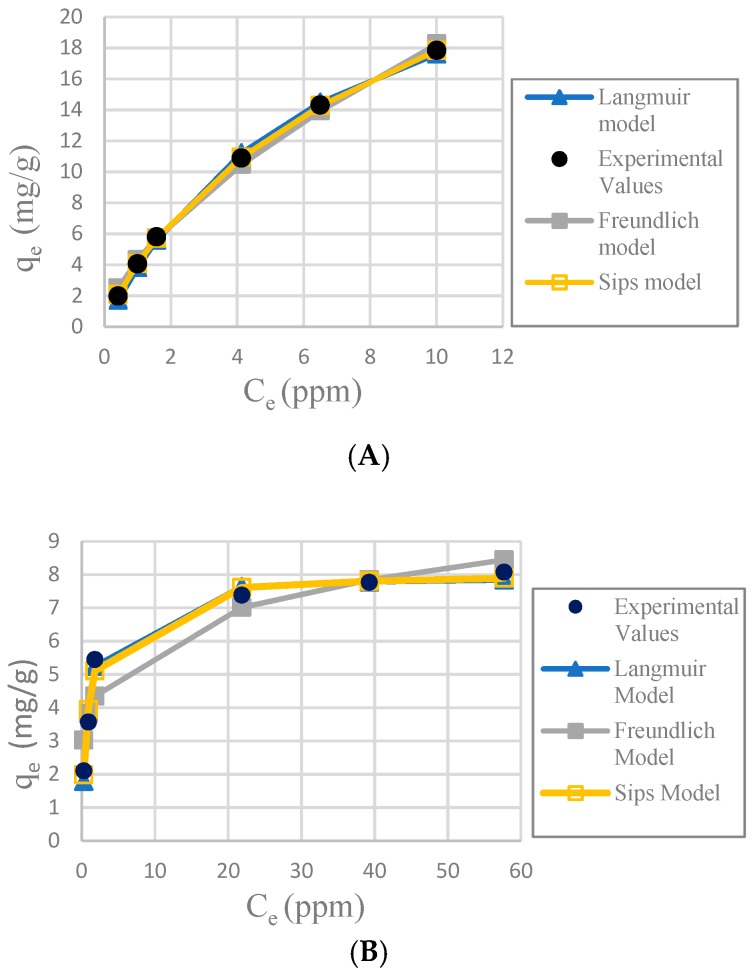

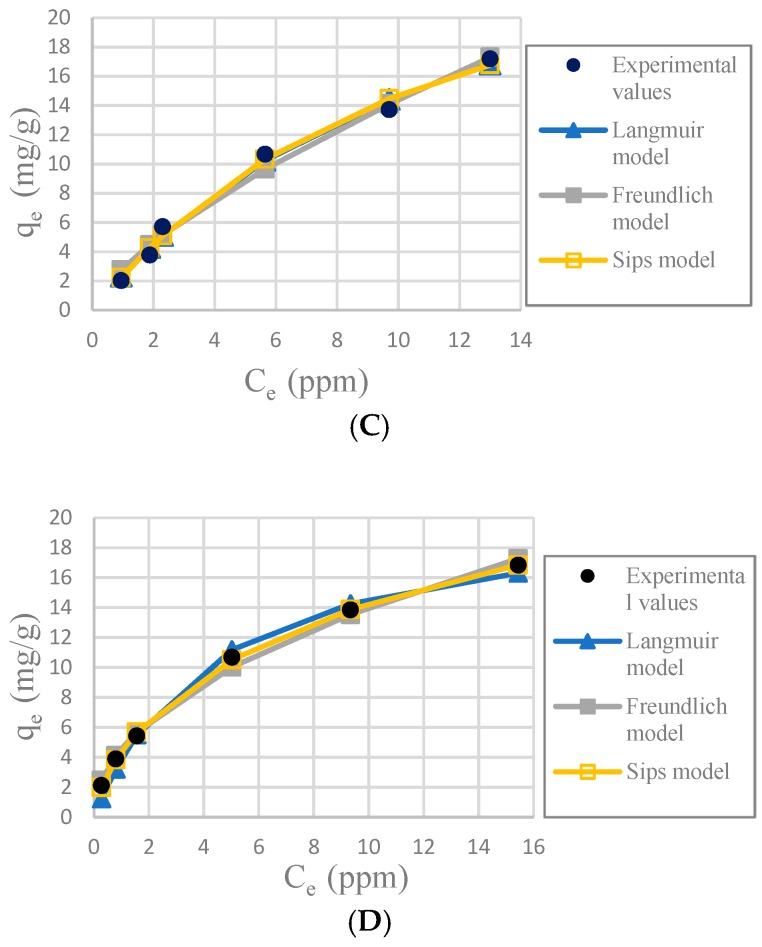
Plots of adsorption isotherms; (**A**): CA-SCGs beads (3:3); (**B**): SCGs; (**C**): CA beads and (**D**): CA-SCGs beads (3:10).

Nonlinear correlation coefficient R^2^, Equation (9) [31]:

(9) $$R^{2}=1-\frac{\sum_{n=1}^{n}{\left[q_{\exp}-q_{\mathrm{model}}\right]}^{2}}{\sum_{n=1}^{n}{\left[q_{e.\exp.n}-q_{\left(\mathrm{average}\right)e.\exp.n}\right]}^{2}}$$

where, q_e.exp_ is the equilibrium sorption capacity found from the batch experiment, q_e.model_ is the prediction from the isotherm model for corresponding to C_e_, and n is the number of observations. The Table 1 shows the results of the adsorption models for each adsorbent. The Figure 7 shows the graphs from the equations of the models for each adsorbent.

### 3.4. Comparison of Isotherms

It was determined that the best fit adsorption isotherm model for all the adsorbents used in this study was Sips, considering the nonlinear correlation coefficient R^2^, relative error ∆*q* and nonlinear chi-square test χ^2^. Sips has better values in the three parameters. The fitness of the other models is more difficult to observe because the three parameters did not always follow the same pattern, but some observations can be made. Freundlich model fit better with CA-SCGs beads (3:3) and CA-SCGs beads (3:10); this is proof of a more heterogenous surface, but it did not fit well for SCGs and CA beads.

The SCGs and CA beads fit better with Langmuir and Sips models. This suggests a more homogeneous surface. The values of the three statistical comparison parameters (R^2^ nonlinear, χ^2^ and ∆*q*) of SCGs and CA beads for the Langmuir and Sips model are similar. In fact, the experimental maximum value of adsorption (mg/g), the Sips q_max_ (mg/g) and the Langmuir q_max_ (mg/g) of SCGs; have a similar value around 8.000 mg/g ± standard deviation (8.079 ± 0.074, 8.113 ± 0.191 and 7.964 ± 0.035; respectively). In the case of CA beads, the Sips q_max_ (mg/g) and the Langmuir q_max_ (mg/g) for the CA beads are not so different; 30.388 ± 0.731 and 33.263 ± 0.922, respectively. Another factor to consider are the values of 1/*n* in the Sips model, a factor related to heterogeneity on the surface, for SCGs and CA beads (0.875 and 1.047, respectively). These values are closer to 1 than the values of the CA-SCGs beads. When this value is closer to one, the Sips equation is reduced to the Langmuir equation. The photos of SEM also suggest a more homogeneous surface for the CA beads. The Langmuir and Sips *q_max_* values for CA beads are lower than other values in the literature, but as it was explained in the introduction, many variables in the synthesis of the beads can affect this value and the only purpose of CA in this study is as a matrix of encapsulation of SCGs.

The values of adsorption capacity obtained by the Sips model and the Langmuir model for the CA-SCGs beads are very different. The *q_max_* values (mg/g) of the Sips model for CA-SCGs (3:3) and CA-SCGs (3:10) beads are 42.059 ± 0.802 and 35.669 ± 0.973, respectively. The *q_max_* values (mg/g) of the Langmuir model for CA-SCGs (3:3) and CA-SCGs (3:10) beads are 29.266 ± 0.683 and 20.921 ± 0.222, respectively. Although the experimental results of the isotherms show a better correlation with the Sips model, the results of the Langmuir model cannot be ignored in the analysis of the isotherms, especially in the case of the CA-SCGs (3:10) beads. First, Figure 6 (The effect of different doses of beads in a solution of 20 ppm Cu^2+)^ shows that the smallest amount of CA-SCGs (3:10) beads in the solution of 20 ppm Cu^2+^ has a diminished capacity of adsorption than the same amount of CA beads in the same solution, in spite of the CA-SCGs (3:10) beads having a somewhat higher adsorption capacity than the CA beads according the Sips model used for the analysis of the isotherms (35.669 mg/g ± 0.973 mg/g versus 30.388 mg/g ± 0.731 mg/g). In the same figure, it can be seen that while the amount of CA-SCGs (3:10) beads is increasing, its capacity of adsorption equals and exceeds the capacity of adsorption of the CA beads. Using the lowest amount of adsorbent in Figure 6, would be equivalent to a concentration of 400 ppm of adsorbate, that is out of the range of concentrations of the isotherms in this study (10 ppm to 100 ppm Cu^2+^). The parameters of the Sips model are dependent of the level of concentration of adsorbate.

The CA-SCGs (3:3) beads have a good fitting with the Langmuir model. On the other hand, the CA-SCGs (3:10) beads did not have a good fitting with this model. Nevertheless, both types of beads show similar fitting with the Freundlich model (Table 1). This fact and other observations in this study such as the photos of SEM, suggest a more heterogenous than homogenous surface for the CA-SCGs (3:3) beads. The differences between the Langmuir model and the Sips model for the CA-SCGs beads can be the product of the heterogeneous surface of these beads. This heterogeneity is supported by the better fitting with the Freundlich model of these beads in comparison with SCGs and CA beads. The SCGs and the CA beads did not have a good fitting with the Freundlich model. This is seen especially in the similar large values of ∆*q* (%) and χ^2^ for SCGs and the CA beads using the Freundlich model (Table 1). Also, as it was mentioned before, both adsorbents have a value of 1/*n* in the Sips model closer to 1. Another reason to support the idea of a heterogeneous surface is the combination of amino functional groups and lipids from SCGs with the structure of CA in the CA-SCGs beads, as was mentioned in the FT-IR characterization.

**Figure 8:**
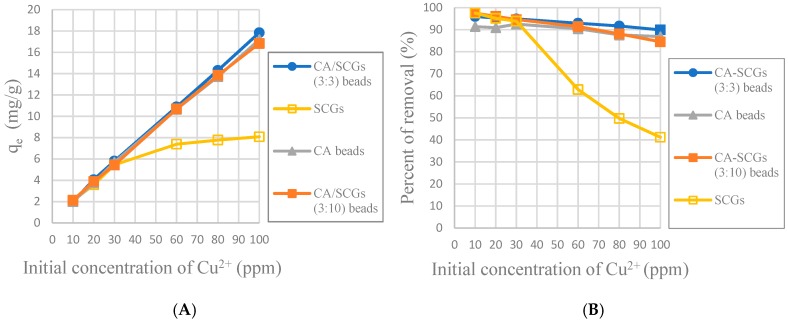
Plots of the amounts absorbed of Cu^2+^ at equilibrium (24 h) at each initial concentration of Cu^2+^; (**A**): Experimental amounts of Cu^2+^ adsorbed at equilibrium (q_e_); (**B**): Percentages of removal at equilibrium (%).

The CA-SCGs (3:3) beads have larger values of experimental amounts of Cu^2+^ adsorbed at equilibrium (q_e_) and percentages of removal (%) than the CA beads in the six initial concentrations used in this study as seen in Figure 8. It is proof that in the range of concentration of Cu^2+^ used in the isotherms of this study (10 ppm to 100 ppm Cu^2+^), the CA-SCGs (3:3) beads have the largest adsorption capacity as indicated by the Sips model.

The maximum adsorption capacity value of the Sips model was 42.050 mg/g ± 0.802 mg/g for the CA-SCGs (3:3) beads. Other materials encapsulated in Calcium Alginate (CA) have shown higher capacities of adsorption such as 60.2 mg/g with graphene oxide [19], 60.0 mg/g with magnetic sorbents [32], and 53.6 mg/g with kaolin [23], but are not considered pollutants. 

The spent coffee grounds reach the saturation at 8 mg/g. This result could be compared with the result obtained by Dávila-Guzmán, N. E et al. [18]. The result of that study was 0.216 mmol/g, equivalent to 13.72 mg/g with a pH of 4.5. The pH used in this study was 4.0. At a lower pH, H^+^ is competing with Cu^2+^ for the adsorption sites. This could be a reason for the lower value of adsorption in our study. Other waste of natural materials such as banana and orange peels have an adsorption of 4.75 mg/g and 3.65 mg/g, respectively [33]. The adsorption of a natural component such as lignin is 22.88 mg/g [34].

The mechanism for adsorption by SCGs suggested by the study by Dávila-Guzmán, N. E et al. is similar to the mechanism suggested by Papageorgiou, S. K. et al. [15] for the adsorption by the CA beads. This mechanism of divalent metal adsorption on CA beads is dominated by ion exchange involving mainly the carboxyl groups present in the alginate molecule, with hydroxyl groups playing a secondary role. SCGs have a lesser amount of carboxyl groups, it could be the reason of the lower adsorption capacity by SCGs but it cannot explain the better affinity and kinetics of SCG below of the saturation level of 8 mg/g. A possible explanation is that not only the carboxylic groups play a major role in the adsorption by SCGs but other functional groups such as phenol groups among others. Alginate has a homogeneous composition of β-D mannuronic Acid(M) and α-L-glucuronic acid (G), where the carboxylic groups are omnipresent in the adsorption sites, and the ion exchange between calcium and copper is necessary for the adsorption. SCGs have a more heterogeneous composition and lignin is present in a significant amount in SCGs. The main acidic compounds of the SCGs analyzed in the study of Pujol, D. et al. [25] are phenolic groups which are mostly located in lignin. The phenolic groups are weakly acidic. The protonated phenolic group is not a particular good ligand for metals cations, but once deprotonated, an oxygen center is generated with a high charge density. The pKa value of the most of phenols is in the region of 9.0–10.0 but in the presence of cations such as Fe^3+^ or Cu^2+^, the proton is displaced at much lower pH values. The phenoxide group favors interaction with cations of high charge density, e.g., Fe^3+^, Fe^2+^, Cu^2+^, and Zn^2+^. Polyphenols do not chelate alkali and alkaline earth cations such as Na^+^, K^+^, and Ca^2+^ [35]. The fact that polyphenols do not chelate Ca^2+^ means that an ion exchange between Ca^2+^ and Cu^2+^ is not expected if the adsorption of Cu^2+^ is by polyphenols. The mechanism would be deprotonation of the phenolic groups and the later binding of Cu^2+^. Calcium is a metal found in large amounts in SCGs relative to other metals. Calcium ions were released during the adsorption of Cu^2+^ by SCGs in the study by Dávila-Guzmán, N. E et al. [18]. This could suggest that the model of carboxylic groups releasing Ca^2+^ ions during the adsorption of Cu^2+^ could be correct because the phenolic groups cannot be bonded to Ca^2+^. Nevertheless, a greater amount of H^+^ ions were released during the same adsorption. This release could be product of the carboxylic groups but also of the phenolic groups. The carboxylic groups in SCGs could be part of hemicellulose and other components of SCGs but they are not ubiquitous in the structure of SCGs as in the case of CA. Xueyan Guo et al. studied the adsorption of heavy metals by lignin and concluded that this material has two types of adsorption sites: carboxylic groups and phenolic groups but the phenolic groups have higher affinity to metal ions [34].

Melanoidins, another component of SCGs, are polymeric macromolecules formed by interactions between carbohydrates and compounds with a free amino group during the roasting process. They have a chelating ability. The mechanism of chelation of melanoidins is not clearly understood because the purification and separation of melanoidins is a complex process. Takenaka, M. et al. separated and characterized a metal chelating substance in coffee using zinc as metal [36]. The results of this study suggested that the substance was a melanoidin-like polymer formed by the decomposition and polymerization of sugars, amino acids and phenolics. The latter study concluded that the metal-chelating activity of this substance may have been due to phenolic residues. This supports the idea that the adsorption by SCGs is different from the adsorption by CA. Functional groups such as the phenolic groups, that are absent in CA, may have an important role in the adsorption of metals by SCGs. The adsorption of metals by SCGs is not only due to the carboxylic groups and the ion exchange between Ca^2+^ and Cu^2+^. SCGs may have less adsorption sites, it is reflected in the reduced adsorption capacity. However, these adsorption sites could have more affinity to Cu^2+^, it is reflected in the kinetics. These differences in the adsorption by the beads were reflected according to the composition of the beads.

### 3.5. Adsorption Kinetics

The effect of the contact time during the adsorption of Cu^2+^ ions by 0.5g of adsorbent in 0.1 L of solution was investigated with initial concentrations between 10 ppm to 100 ppm of Cu^2+^. The pseudo-second-order equation was used because it showed the best fit with the results (R^2^ > 0.979). This fact indicated the adsorption process of the Cu^2+^ ions was complex for CA beads, CA-SCGs beads and SCGs. More than one mechanism is involved. The pseudo-second-order linear equation was used to evaluate the data, Equation (10) [37]:

*t*/*q_t_* = 1/(*k*_2_*q_e_*^2^) + *t*/*q_e_* (10)

where *k*_2_ (g/ mg-min) is the rate constant of pseudo-second-order adsorption, *q_e_* (mg/g) is the amount of Cu^2+^ ions at equilibrium, and *q_t_* (mg/g) is the amount of Cu^2+^ ions adsorbed at time t (min). Making a graph *t/q_t_* versus *t*, the slope and the intercept give the values of *k*_2_ and the *q_e_* calculated during the adsorption of Cu^+2^ ions at a specific initial concentration of Cu^2+^.

Another kinetic parameter is *h* (mg g^−1^min^−1^). This parameter is the initial adsorption rate when *t* → 0. This parameter can be calculated by the following equation, Equation (11) [37]:

*h* = *k*_2_*q_e_*^2^ (11)

**Figure 9:**
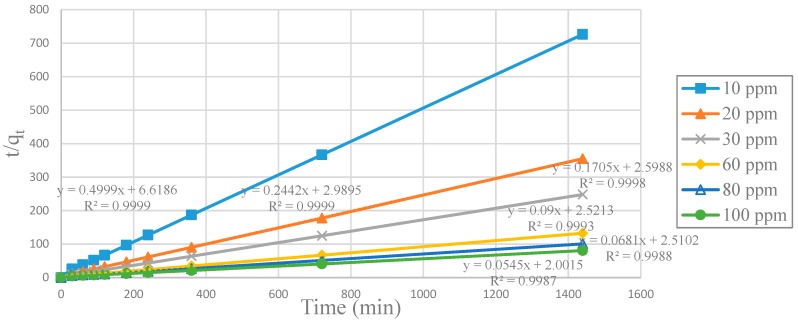
Plot of pseudo-second-order linear equations for CA-SCGs (3:3) beads for each initial concentration of Cu^2+^.

Graphs of *t*/*q_t_* versus *t* such as the plot in Figure 9 were made for SCGs, CA beads, CA-SCGs (3:3) beads and CA-SCGs (3:10) beads. Values of *k*_2_, *q_e_* calculated and *h* were obtained for each initial concentration of Cu^2+^ from each graph.

SCGs is the absorbent with the highest kinetic rate constants (*k*_2_) and initial adsorption rate constants (*h*), at all initial concentrations of Cu^2+^. In the same way, the CA beads have the lowest values of *k*_2_ and *h* at all initial concentrations of Cu^2+^. The CA-SCGs beads have much higher kinetic parameters than the CA beads (Table 2).

**Figure 10:**
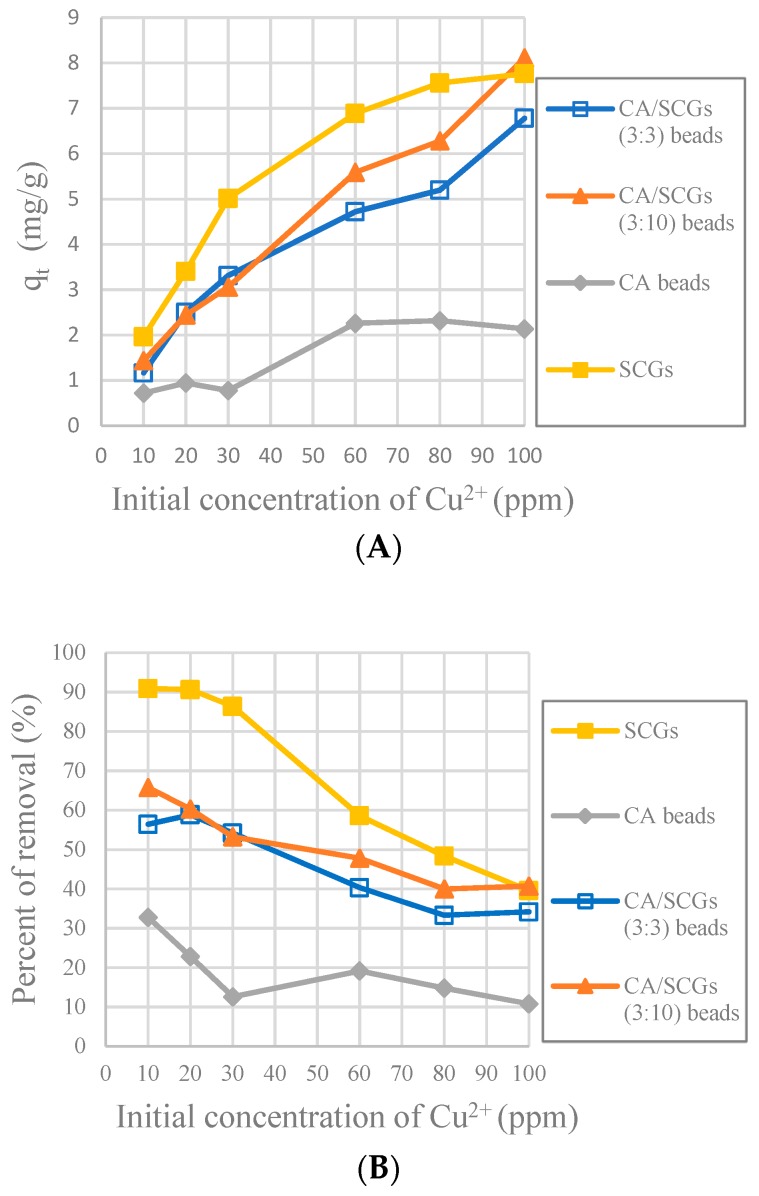
Plots of the amounts absorbed of Cu^2+^ at 30 min at each initial concentration of Cu^2+^, (**A**): Experimental amounts of Cu^2+^ adsorbed at 30 min (q_t_), (**B**): Percentages of removal at 30 min (%).

Figure 10 shows that SCGs have the maximum adsorption at the most initial concentrations of Cu^2+^ in the first 30 min, but the adsorption by SCGs is limited close to its experimental maximum value of adsorption of 8 mg/g. The CA-SCGs (3:3) beads and CA-SCGs (3:10) beads have a much better adsorption than the CA beads in the first 30 min. At 30 min, the adsorbent with the largest q_t_ are the CA-SCGs (3:10) beads with a q_t_ of 8.111 mg/g at an initial concentration of Cu^2+^ of 100 ppm. 

**Figure 11:**
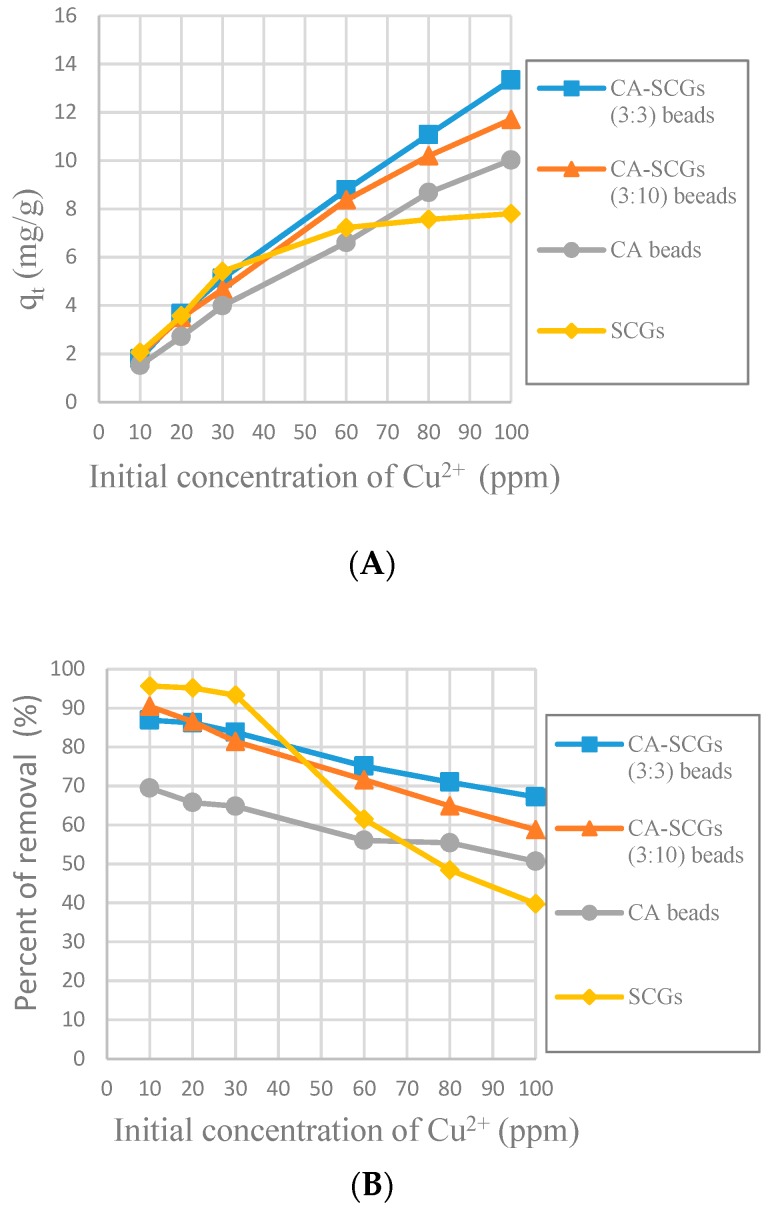
Plots of the amounts absorbed of Cu^2+^ at 120 min at each initial concentration of Cu^2+^, (**A**): Experimental amounts of Cu^2+^ adsorbed at 120 min (q_t_); (**B**): Percentages of removal at 120 min (%).

The purpose of the plots in Figure 11 is to see the adsorption performance of the CA-SCGs beads beyond the equilibrium time of SCGs and the level of saturation of SCGs at 8 mg/g. At 120 min, the CA-SCGs beads still have a better adsorption performance than the CA beads. The adsorption by the CA-SCGs (3:3) beads maintains an almost linear correlation with the initial concentration of Cu^2+^ beyond of the level of 8 mg/g.

In Figure 8, it was seen that even at 24 h, the CA-SCGs (3:3) beads have better adsorption than the CA beads. With an initial concentration of 100 ppm of Cu^2+^, the CA-SCGs (3:3) beads have a q_e_ of 17.839 mg/g ± 0.258 mg/g, more than the q_e_ of the CA beads of 17.184 mg/g ± 0.384 mg/g, the q_e_ of the CA-SCGs (3:10) beads of 16.828 mg/g ± 0.397mg/g and much more than the q_e_ of SCGs of 8.079 mg/g ± 0.074 mg/g.

**Figure 12:**
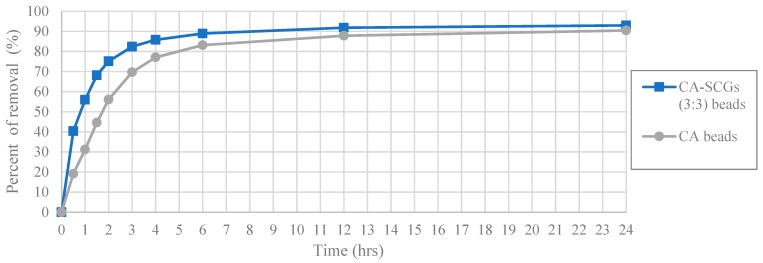
Plot of Percent of removal vs Time at initial concentration of 60 ppm of Cu^2+^, CA and CA-SCGs (3:3) beads.

The worst results of adsorption by CA/SCG beads are expected at concentrations of 60 ppm or more because the spent coffee grounds achieve the saturation level of 8 mg/g at these concentrations as seen in the Figure 8. The plot in Figure 12 is an example of how the CA beads need more time to achieve the same percentages of removal than the CA-SCGs (3:3) beads. The CA-SCGs (3:3) beads achieve 55.95% (6.550 mg/g) at 1 h and the CA beads achieve 56.05% (6.612 mg/g) at 2 h. The CA-SCGs (3:3) beads achieve 75.09% (8.790 mg/g) at 2 h and the CA beads achieve 77.08% (9.092 mg/g) at 4 h. The CA beads need double the time to achieve similar percentages of removal than the CA-SCGs (3:3) beads during the first hours of the experiment, at an initial concentration of 60 ppm Cu^2+^.

**Figure 13:**
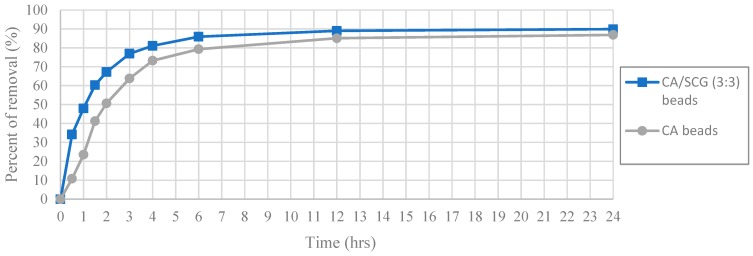
Plot of Percent of removal vs Time at initial concentration of 100 ppm of Cu^2+^, CA and CA-SCGs (3:3) beads.

The same pattern is repeated at the highest initial concentration of 100 ppm of Cu^2+^ (Figure 13). The CA-SCGs (3:3) beads achieve 47.97% (9.518 mg/g) at 1 h and the CA beads achieve 50.67% (10.024 mg/g) at 2 h. The CA-SCGs (3:3) beads achieve 67.21% (13.336 mg/g) at 2 h and the CA beads achieve 73.23% (14.488 mg/g) at 4 h. Although the CA beads achieve a little more removal of Cu^2+^ relative to the CA-SCGs (3:3) beads at this higher concentration of Cu^2+^.

**Figure 14:**
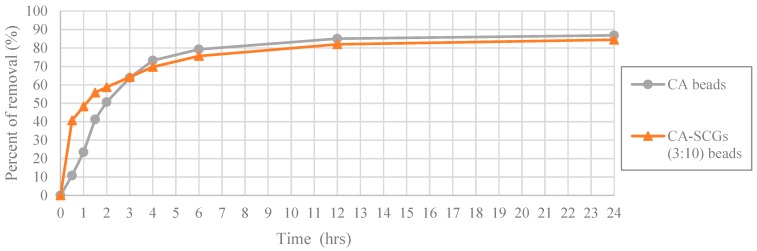
Plot of Percent of removal vs Time at initial concentration of 100 ppm of Cu^2+^, CA and CA-SCGs (3:10) beads.

In Figure 14, at the highest initial concentration of 100 ppm of Cu^2+^; the CA-SCGs (3:10) beads and the CA beads achieve percentages of removal of 40.72% (8.111 mg/g) and 10.80% (2.136 mg/g) at 0.5 h, respectively. The CA-SCGs (3:10) beads achieve almost four times the percent of removal of Cu^2+^ by the CA beads at this time. The CA beads achieve 41.24% (8.160 mg/g) at 1.5 h, the triple of time needed by the CA-SCGs (3:10) beads to achieve a similar percent of removal. However, the speed of adsorption of the CA-SCG (3:10) beads starts to slow down as more adsorbate is removed from the solution. The CA beads and the CA-SCGs (3:10) beads achieve similar percentages of removal at 3 h, 63.81% (12.625 mg/g) and 64.20% (12.787 mg); respectively. The excess of SCGs used in the synthesis of CA-SCGs (3:10) beads improves the kinetics of adsorption at the beginning of the experiment, but it affects the capacity of adsorption of the CA-SCGs (3:10) beads as more adsorbate is removed. This can be due to the saturation of the SCGs in the beads. An interesting fact is that the CA-SCGs (3:10) beads are composed mostly of SCGs, not CA. When the bead was formed, the SCGs used in the synthesis of the bead were trapped inside the bead. Nevertheless, the capacity of adsorption of the CA-SCGs (3:10) beads is not so low as the capacity of adsorption of SCGs alone (8.079 mg/g), and it is similar to the capacity of adsorption of the CA beads at this initial concentration of 100 ppm of Cu^2+^. The values of q_e_ of the CA beads and the CA-SCGs (3:10) beads are 17.184 mg/g ± 0.384 mg/g and 16.828 mg/g ± 0.397 mg/g, respectively. The percentages of removal were 86.86% and 84.49%. Possibly, the encapsulation of the SCGs in a matrix of CA changes the equilibrium of adsorption of Cu^2+^ by the SCGs. The interaction between SCGs and CA during the formation of the CA/SCG beads deserves further investigation to see why the capacity of adsorption of the CA/SCG (3:10) beads and the CA beads is similar at that range of concentrations, in spite that the CA/SCG (3:10) beads have an excess of SCGs and the SCGs have lower capacity of adsorption than CA at the same range of concentrations.

According the pseudo-second-order adsorption kinetic parameters of *k_2_* and *h* (Table 2), the CA-SCGs beads have a faster adsorption rate of Cu^2+^ than the CA beads. This fact could be seen experimentally in Figure 10 and Figure 11. This adsorption rate is maintained beyond the equilibrium time and the level of saturation of SCGs at 8 mg/g (Figure 11). The CA-SCGs (3:3) beads exhibit the best adsorption capacity through the 24 h of the study at all initial concentrations of Cu^2+^ (Figure 8) and keep the fastest rate of adsorption through the same time (Figure 12 and Figure 13).

## 4. Conclusion 

SCGs exhibit the largest adsorption rate at the beginning of the adsorption test, achieving equilibrium in minutes. However, their rate of adsorption of Cu^2+^ is limited at the level of maximum adsorption capacity of 8 mg/g; beyond this limit, the adsorption by this adsorbent is halted. The results suggest that SCGs have a lesser amount of adsorption sites than CA but these adsorption sites have a greater affinity to copper. The CA-SCGs (3:3) beads with a proportion of alginate/spent-coffee-grounds of 1:1 show the best qualities as adsorbents in this study. The adsorption isotherm models suggest that the adsorption by the CA-SCGs (3:3) beads is a different process than the adsorption by the CA beads, likewise the adsorption process by the CA-SCGs (3:3) beads is not only due to the CA component of these beads. Also, the SEM photos show that they have totally different surfaces. The adsorption by the CA-SCGs (3:3) beads is faster than the adsorption by the CA beads and the maximum adsorption capacity at equilibrium is not diminished due its content of SCGs. The addition of an excess of SCGs in the synthesis of the CA-SCGs (3:10) beads can affect the maximum adsorption capacity at equilibrium of these beads in comparison with the CA beads, but not significantly; even though these beads are composed mostly of SCGs, not CA. This is according to the experimental results at initial concentrations of 10 ppm to 100 ppm of Cu^2+^. The best adsorbent in this study, CA-SCGs (3:3) beads, do not have the limitation of adsorption capacity of the SCGs alone and have a faster adsorption rate of Cu^2+^ than the CA used in this study to encapsulate the SCGs. The synthesis of the CA-SCGs (3:3) beads can help in the removal of heavy metals in water and recycle the SCGs, which are considered pollutants in the landfills.

## Figures and Tables

**Table 1:** Adsorption isotherm constants and statistical comparison values for Langmuir, Freundlich and Sips models.

**Langmuir Beads**	**SCGs**	**CA Beads**	**CA/SCGs (3:3) Beads**	**CA/SCGs (3:10)**
*q_max_* (mg/g)	7.964	33.263	29.266	20.921
*b*_L_ (L/mg)	1.079	0.078	0.150	0.228
R^2^ (nonlinear)	0.983	0.989	0.997	0.985
χ^2^	0.011	0.243	0.070	0.347
∆*q* (%)	5.982	8.493	4.673	11.762
**Freundlich Beads**	**SCGs**	**CA Beads**	**CA/SCGs (3:3) Beads**	**CA/SCGs (3:10)**
*K_f_* (mg/g)(L/mg)^(1/n)^	3.891	2.869	4.324	4.592
1/*n*	0.190	0.700	0.624	0.483
R^2^ (nonlinear)	0.902	0.981	0.994	0.993
χ^2^	0.679	0.581	0.185	0.139
∆*q* (%)	13.576	13.215	7.038	6.283
**Sips Beads**	**SCGs**	**CA Beads**	**CA/SCGs (3:3) Beads**	**CA/SCGs (3:10)**
*q_max_* (mg/g)	8.113	30.388	42.059	35.669
*b*_S_ (L/mg)	1.024	0.084	0.107	0.140
1/*n*	0.875	1.047	0.834	0.676
R^2^ (nonlinear)	0.985	0.989	0.999	0.999
χ^2^	0.008	0.217	0.006	0.025
∆*q* (%)	4.682	7.716	1.262	2.448

**Table 2:** Pseudo-second-order adsorption kinetic parameters.

Adsorbent	R^2^	*q_e_* Experimental (mg/g)	*q_e_* Calculated (mg/g)	*k*_2_ (g/mg-min)	*h* (mg/g-min)
CA-SCGs beads (3:3)	0.9999	1.9834	2.0002	0.0378	0.1511
10 ppm Cu^2+^					
CA-SCGs beads (3:3)	0.9999	4.0550	4.0952	0.0199	0.3345
20 ppm Cu^2+^					
CA-SCGs beads (3:3)	0.9998	5.8016	5.8651	0.0112	0.3848
30 ppm Cu^2+^					
CA-SCGs beads (3:3)	0.9993	10.8804	11.1111	0.0032	0.3966
60 ppm Cu^2+^					
CA-SCGs beads (3:3)	0.9988	14.3024	14.6843	0.0018	0.3983
80 ppm Cu^2+^					
CA-SCGs beads (3:3)	0.9987	17.8392	18.3486	0.0015	0.4996
100 ppm Cu^2+^					
CA beads	0.9981	2.0064	2.0687	0.0129	0.0554
10 ppm Cu^2+^					
CA beads	0.9959	3.7600	3.9216	0.0049	0.0759
20 ppm Cu^2+^					
CA beads	0.9851	5.7000	6.0753	0.0021	0.0770
30 ppm Cu^2+^					
CA beads	0.9934	10.6664	11.3122	0.0012	0.1475
60 ppm Cu^2+^					
CA beads	0.9898	13.7208	14.6843	0.0008	0.1756
80 ppm Cu^2+^					
CA beads	0.9795	17.1840	18.7617	0.0005	0.1732
100 ppm Cu^2+^					
CA-SCGs beads (3:10)	0.9999	2.1224	2.1363	0.0482	0.2199
10 ppm Cu^2+^					
CA-SCGs beads (3:10)	0.9999	3.9018	3.9246	0.0206	0.3178
20 ppm Cu^2+^					
CA-SCGs beads (3:10)	0.9998	5.4278	5.4945	0.0101	0.3050
30 ppm Cu^2+^					
CA-SCGs beads (3:10)	0.9994	10.7156	10.8814	0.0031	0.3724
60 ppm Cu^2+^					
CA-SCGs beads (3:10)	0.9990	13.8558	14.1443	0.0019	0.3772
80 ppm Cu^2+^					
CA-SCGs beads (3:10)	0.9984	16.9820	17.2716	0.0013	0.3919
100 ppm Cu^2+^					
SCGs	1.0000	2.1053	2.1075	0.2250	0.9992
10 ppm Cu^2+^					
SCGs	1.0000	3.5742	3.5765	1.0753	13.7552
20 ppm Cu^2+^					
SCGs	1.0000	5.4495	5.4555	0.3621	10.7759
30 ppm Cu^2+^					
SCGs	1.0000	7.3853	7.4019	0.1610	8.8183
60 ppm Cu^2+^					
SCGs	1.0000	7.7725	7.7882	0.1398	8.4818
80 ppm Cu^2+^					
SCGs	0.9995	8.0791	8.0386	0.0228	1.4725
100 ppm Cu^2+^					
